# The degradation of airway tight junction protein under acidic conditions is probably mediated by transient receptor potential vanilloid 1 receptor

**DOI:** 10.1042/BSR20130087

**Published:** 2013-10-31

**Authors:** Rui Xu, Qi Li, Jia Zhou, Xiang-dong Zhou, Juliy M. Perelman, Victor P. Kolosov

**Affiliations:** *Department of Respiratory Medicine, the Second Affiliated Hospital, Chongqing Medical University, Chongqing 400010, China; †Department of Respiratory Medicine, the First Affiliated Hospital of Chongqing Medical University, Chongqing 400016, China; ‡Far Eastern Scientific Center of Physiology and Pathology of Respiration, Russian Academy of Medical Sciences, Blagoveschensk 675000, Russia

**Keywords:** acidic stress, airway, epithelial tight junction, TRPV1, BAPTA/AM, 1,2-bis-(*o*-aminophenoxy)ethane-*N*,*N*,*N*',*N*'-tetra-acetic acid tetrakis(acetoxymethyl ester), COPD, chronic obstructive pulmonary disease, DMEM, Dulbecco’s modified Eagle’s medium, EBC, exhaled breath condensate, fluo-3/AM, fluo-3/acetoxymethyl, MAGUK, membrane-associated guanylate kinase, MTT, 3-(4,5-dimethylthiazol-2-yl)-2,5-diphenyl-2*H*-tetrazolium bromide, TER, trans-epithelial electrical resistance, TJs, tight junctions, TRPV, transient receptor potential vanilloid, ZO, zonula occluden

## Abstract

Acidic airway microenvironment is one of the representative pathophysiological features of chronic inflammatory respiratory diseases. Epithelial barrier function is maintained by TJs (tight junctions), which act as the first physical barrier against the inhaled substances and pathogens of airway. As previous studies described, acid stress caused impaired epithelial barriers and led the hyperpermeability of epithelium. However, the specific mechanism is still unclear. We have showed previously the existence of TRPV (transient receptor potential vanilloid) 1 channel in airway epithelium, as well as its activation by acidic stress in 16HBE cells. In this study, we explored the acidic stress on airway barrier function and TJ proteins *in vitro* with 16HBE cell lines. Airway epithelial barrier function was determined by measuring by TER (trans-epithelial electrical resistance). TJ-related protein [claudin-1, claudin-3, claudin-4, claudin-5, claudin-7 and ZO-1 (zonula occluden 1)] expression was examined by western blotting of insoluble fractions of cell extraction. The localization of TJ proteins were visualized by immunofluorescent staining. Interestingly, stimulation by pH 6.0 for 8 h slightly increased the epithelial resistance in 16HBE cells insignificantly. However, higher concentration of hydrochloric acid (lower than pH 5.0) did reduce the airway epithelial TER of 16HBE cells. The decline of epithelial barrier function induced by acidic stress exhibited a TRPV1-[Ca^2+^]_i_-dependent pathway. Of the TJ proteins, claudin-3 and claudin-4 seemed to be sensitive to acidic stress. The degradation of claudin-3 and claudin-4 induced by acidic stress could be attenuated by the specific TRPV1 blocker or intracellular Ca^2+^ chelator BAPTA/AM [1,2-bis-(*o*-aminophenoxy)ethane-*N*,*N*,*N*',*N*'-tetra-acetic acid tetrakis(acetoxymethyl ester)].

## INTRODUCTION

The airway epithelium of human respiratory mucosa acts as the first physical barrier against the inhaled substances and pathogens [1,2]. The epithelial barrier function is highly related with the integrity of TJs (tight junctions). As termed ‘barrier function’, epithelial TJs, most apically located of the intercellular junctional complexes, inhibit solute, water and inflammatory factors through the paracellular space [3,4]. Also, TJs separate the apical domain from basolateral and maintain the cell polarity in human airway epithelium [5]. TJs are formed by integral membrane proteins claudins, occludin, JAMS (junctional adhesion molecules) and the peripheral membrane proteins ZOs (zonula occludens). Claudins are considered to be the most important components of the TJs at the interface of the basolateral and apical membranes of polarized epithelial cells. They determine the barrier properties of the cell–cell contact between two neighbouring epithelial cells and regulate the paracellular permeability. Of the claudin family members, claudin-3, claudin-4 and claudin-5 were detected in rat type II alveolar epithelial cells. In addition, claudin-2 is also expressed in the human lung cell line A549 [6–8]. ZO-1, the member of MAGUK (membrane-associated guanylate kinase) family, is associated with polymerization of claudins. ZO-1 locates between occludin and cytoskeletal proteins and is thought to be associated with paracellular permeability in airway epithelium [9]. According to recent studies, cytokines increased airway permeability by attenuating ZO-1 from cell surface, which could be partially blocked by EGFR (epidermal growth factor receptor) or MAPK (mitogen-activated protein kinase) inhibitor. [9].

Normally the pH of human airway ranges at about 7.7 according to the examination of EBC (exhaled breath condensate) [3,10,11]. However, acidic respiratory microenvironment occurs in a majority of airway inflammatory diseases, such as asthma, COPD (chronic obstructive pulmonary disease) exacerbation, bronchiectasis and cystic fibrosis [12]. Based on the acidification of EBC, acute asthma exhibits an airway pH at 5.23 in average [13]. COPD patients have a lower airway pH at about 7.21 compared with an average of 7.5 in health controls. However, the airway pH is much lower in COPD exacerbation. Airway acidification is high related with the severity of airway inflammatory diseases and efficacy of the therapy [14,15]. There is evidence that acidification elicits instability of the epithelial TJ complex in both *in vivo* and *in vitro* studies [16,17]. However, the exact mechanisms are still indecisive.

The TRP (transient receptor potential) family of proteins is currently under intense investigation in health and disease because these ion channels have been recognized to sense a vast range of stimuli. TRPV (transient receptor potential vanilloid) 1, a member of the vanilloid subtype of the TRP family of non-selective cation channels, can be activated by low extracellular pH. According to the previous studies, TRPV1 channels can be directly activated by low extracellular pH (<6.0) or moderate noxious temperature between 42 and 53°C [18]. It is evidence that the up-regulation of TRPV1 channels in mucous epithelial cells is high related with inflammatory diseases as asthma, COPD and allergic rhinitis [18,19]. In our previous investigation, TRPV1 was demonstrated to be expressed in 16HBE cells and responsible for the Ca^2+^ influx in airway epithelial cells reacted to acidic stress [20]. Based on the findings that the degradation of TJs induced by acidification was probably relied on the concentration of intercellular Ca^2+^, we hypothesized a TRPV1 associated mechanism for the degradation of TJs induced by acid stress in airway epithelium.

## MATERIALS AND METHODS

### Materials

DMEM (Dulbecco's modified Eagle's medium), capsaicin, capsazepine, were purchased from Sigma. FBS was purchased from Invitrogen. The antibodies: rabbit polyclonal antibody to ZO1, rabbit polyclonal antibody to claudin-1, rabbit polyclonal antibody to claudin-3, mouse monoclonal antibody to claudin-4, rabbit polyclonal antibody to claudin-5 and rabbit polyclonal antibody to claudin-7 were purchased from Abcam. The internal reference and second antibodies were purchased from Zhongshan Goldenbridge Biotechnology.

### Cell culture

Human 16HBE cells were purchased from Guangzhou Respiratory Institute (Guangzhou, China). 16HBE cells are SV40 (simian virus 40) virus-transformed, immortalized human bronchial epithelial cells. Cells were propagated in DMEM (adjusted the pH to 7.4) supplemented with 10% (v/v)FBS, 50 μm/ml penicillin and 100 μg/ml streptomycin in a 37°C, 5% (v/v) CO_2_ incubator. The 16HBE cells were plated in 6×60 mm culture dishes at a density of ~2×10^6^ /ml and cultured in a 37°C, 5% CO_2_ incubator to allow the cells to attach.

### Preparation acidification stress

Acidic stress is a commonly pathophysiologic condition used to study respiratory diseases in humans and laboratory animals [21]. To investigate the relationship between acidification and the permeability of airway epithelium *in vitro*, the pH values of DMEM culture medium was adjusted by hydrochloric acid to the following different gradients: pH=7.4, 7.0, 6.0, 5.0 and 4.0. Sodium chloride was selected as a modifier to achieve a same concentration of Cl^−^[22]. Different exposure times (0, 4, 8, 12 h) were performed on 16HBE. Cell viability was evaluated by a conventional MTT [3-(4,5-dimethylthiazol-2-yl)-2,5-diphenyl-2*H*-tetrazolium bromide] reduction assay. According to the maximum cell viability and enough exposure time, 8 h was selected as the recommend exposure time.

### Western blot for the expression of TJs

Total membrane proteins were extracted following the protocol of the Membrane and Cytosol Protein Extraction Kit. The insoluble fractions of the cell extractions were loaded onto an SDS/PAGE gel and transferred to PVDF membranes. The PVDF membranes were blocked by 5% (w/v) non-fat dried skimmed milk powder subsequently incubated with primary antibodies for ZO-1 (Abcam, ab59720) at 1:50 dilution, claudin-1 (Abcam, ab15098) at 1:800 dilution, claudin-3 (Abcam, ab15102) at 1:800 dilution, claudin-4 (Abcam, ab15104) at 1:500 dilution, claudin-5 (Abcam, ab53765) at 1:800 dilution, claudin-7 (Abcam, ab27487) at 1:100 dilution respectively overnight. The PVDF membranes were washed by TBST for three times and subsequently incubated with second antibody HRP (horseradish peroxidase) goat anti-rabbit IgG (immunoglobin G) or HRP goat anti-mouse IgG, respectively (Zhongshan Goldenbridge Biotechnology). Protein bands were visualized by enhanced chemiluminescence followed the manufacturer's instruction (Beyo ECL Plus). The intensity of each band was measured by Fluor-S MultiImager and Quantity-One software (Bio-Rad). Protein expression level was normalized to that of internal references.

### Analysis for intracellular Ca^2+^ concentration with a laser scanning confocal microscope

Briefly, 20 μl 16HBE cells suspensions from each group were collected and loaded with 20 μl of 40 μM fluo-3/AM (fluo-3/acetoxymethyl) in dark for 30 min at 37°C. To remove the extracellular fluo-3/AM, the cell suspensions were then washed three times with D-Hank's solution. To determine the concentration of intracellular Ca^2+^ in 16HBE cells, laser scanning confocal microscope analysis was performed with a Leica Sp2 confocal microscope. The parameters were set up to the magnification 40×, the excited light 488 nm, the emission light 515/25 nm, the pinhole 10–40 nm, and the power 30%. Intracellular Ca^2+^ levels of 16HBE cell with green fluorescence were judged by FI (fluorescent intensity) [20]. Data were recorded for the statistical analysis.

### Evaluation of epithelial barrier function of 16HBE cells

Epithelial barrier function was evaluated by TER (transepithelial electrical resistance). 16HBE cells were seeded into transwell inserts at a density of 5×10^5^/well. Cells were cultured for the formation of intercellular adhesion. Cell layer TER was evaluated using the Millicell-ERS system (Millipore Co.) before and after hydrochloric acid treatments [23]. The TER values (Ω×cm^2^) were calculated by the following equation: (TER sample−TER blank)×surface area.

### Cell immunochemistry and laser confocal microscopy

The direct visual observation of TJs was performed using immunochemistry and laser confocal microscopy. 16HBE cells were plated at a density of 1×10^6^/ml in 6-well plates on a glass coverslip in each well. Culture media were refreshed every 2 days. After the formation of cell–cell adhension, the cells were washed three times with PBS and exposed to the acidic culture medium for 8 h, as the ‘preparation’ mentioned above. The cells were fixed with 4% (v/v) paraformaldehyde for 15 min and washed three times with PBS. The cells were then treated by 0.1% (v/v) TritonX-100 for 10 min, and washed again. Followed by blocking in 5% (v/v) goat serum for 60 min, cells were then incubated with primary antibodies for the components of TJs, respectively overnight in a 4°C refrigerator. After washing three times with PBS, the slides were incubated with the secondary antibody, FITC-linked goat anti-rabbit IgG or FITC-linked goat anti-mouse IgG, respectively (Zhongshan Goldenbridge Biotechnology) at 1/50 dilution, for 60 min. The slides were then washed three times with PBS, and incubated in 5% (v/v) propidium iodide for 3 min. After washed again in PBS for five times, the slides were embedded in 50% (v/v) glycerol. 16HBE cells were visualized using a confocal microscope (TCS-SP2, Leica). Representative images were taken with a digital camera and then processed with Adobe Photoshop CS2.

### Statistical analysis

Data were reported as mean±S.D. of six independent experiments. All data were analysed with SPSS 17.0 statistical package. The analysis of one-way ANOVA with SNK-*q* test was used to compare the levels of difference between groups. Statistical significance is indicated where *P*<0.05.

## RESULTS

### MTT assay for cell viability

**Figure 1:**
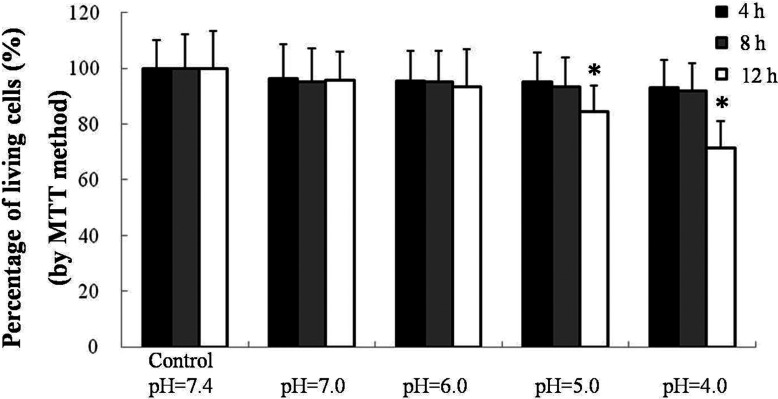
MTT assay for cell viability 16HBE cells propagated in pH=7.4 culture medium were set as negative controls. 16HBE cells were exposed to different concentrations of hydrochloric acid (pH=7.4, 7.0, 6.0, 5.0, 4.0) and for different exposure durations (4, 8, 12 h). Data were represented as mean ± S.D.; *n*=6, *P<0.05 versus control.

To evaluate the cell viability after exposure to different acidic culture medium, conventional MTT assay was performed to evaluate influence of cell viability at low pH. The result demonstrated a significant reduction of cell viability at pH=5.0 and 4.0 for 12 h. Thus, 8 h was selected as the appropriate exposure time (Figure 1).

### Acidification circumstance increases airway epithelial permeability and TJs degradation

**Figure 2:**
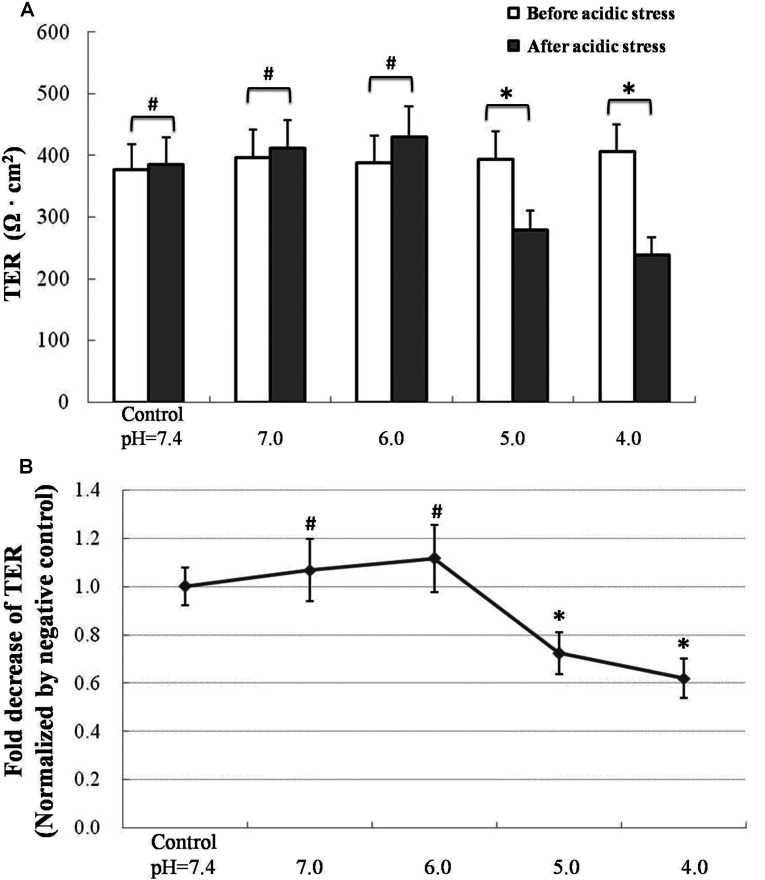
Effect of acidification on airway epithelial barrier function (**A**) 16HBE cells exposed to pH 7.4 culture medium for 8 h were set as negative control. TER values of 16HBE cells were recorded before and after exposure to acidic stress. Values are mean±S.D.; *n*=6. *P<0.05. ^#^*P*>0.05. (**B**) Fold changes of TER values in 16HBE cells after acidic stress. 16HBE cells after exposure to pH 7.4 culture medium for 8 h were set as base line. Values are mean±S.D.; *n*=6. *P<0.05 compared with the base line. ^#^*P*>0.05 compared with the base line.

Airway epithelial function was estimated by TER as described in the experimental section. 16HBE cells exposed in DMEM with the pH of 7.4 was set as the negative control. For statistic analysis, TER values of 16HBE cells after exposure to different acidification culture mediums were normalized by TER of 16HBE cells after exposure to pH 7.4 culture medium. No significant changes were found in TER values of 16HBE cells in pH=7.4 or 7.0 culture medium 8 h later. Interestingly, TER values of 16HBE cells exhibited a slightly increase after exposure to the culture medium with pH=6.0. However, there was no statistical significance. Stimulated with higher acidification (pH=5.0, 4.0) increased the permeability of 16HBE cells. (Figure 2A) Normalized by negative control, TER of 16HBE cells exhibited an insignificant increase after slight acidification (pH=6.0). However, TER values of 16HBE cells were significantly decreased after pH=5.0 or 4.0 acidic stimulation compared with basal level, which indicated a loss of airway epithelial barrier function followed by higher dose of acidic stimulation. Exposed to pH 5.0 culture medium, the TER value of 16HBE cells approximately decreased more than 20% compared with the basal level. Acidification of pH 4.0 induced a nearly 40% reduction of TER values in 16HBE cells (Figure 2B).

### The expression of claudin-3 and claudin-4 in airway epithelium is sensitive to acidic stress

**Figure 3:**
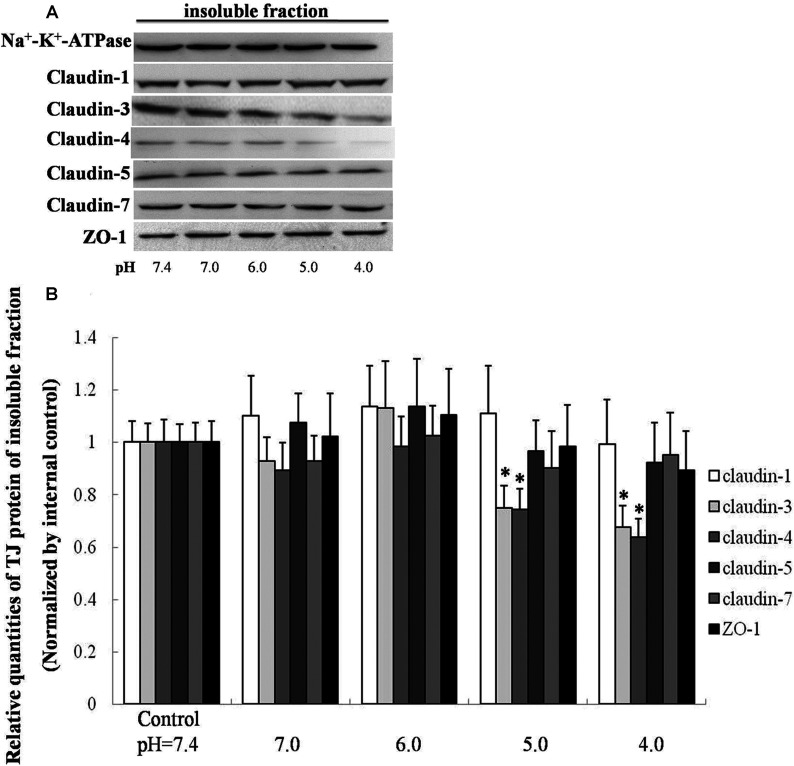
Effect of acidification on TJ proteins (**A**) Cell extractions of insoluble fraction were loaded. 16HBE cells after exposure to pH 7.4 culture medium for 8 h were set as negative controls. (**B**) Relative expression of TJ proteins. The expression levels of TJ proteins in insoluble fracture were normalized to those of negative controls respectively. Values are mean±S.D.; **n**=6. *P<0.05 compared with controls.

**Figure 4:**
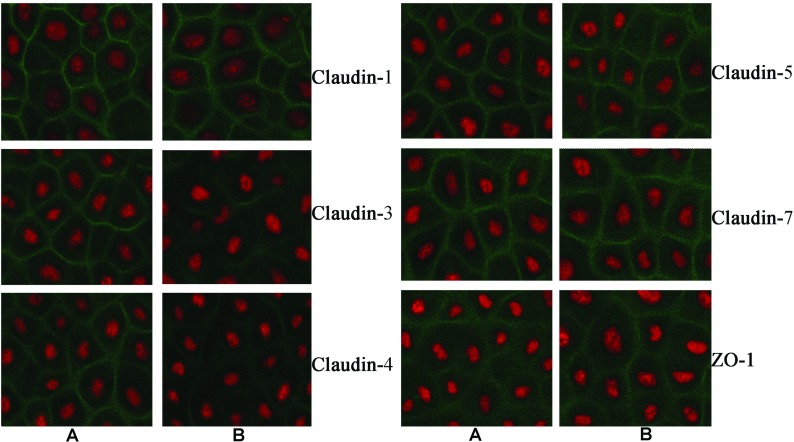
Effect of acid stimulation on the disassembly of TJ protein in 16HBE cells Representative merged photomicrographs obtained by confocal microscopy after immunofluorescence staining. Original magnification×800. (**A**) The representative pictures of 16HBE cells incubated in pH 7.4 DMEM for 8 h. (**B**) The representative pictures of 16HBE cells incubated in pH 5.0 DMEM for 8 h. The results shown are representative of the results obtained in six independent experiments (Colour figure online).

To determine the influence of different degrees of acidification on the expression of TJ proteins, claudin-1, claudin-3, claudin-5, claudin-7 and ZO-1 were tested in membranal extracts. Acidification of the culture medium changed claudin-3 and claudin-4 levels in insoluble fraction. However, other proteins of TJs in 16HBE cells as claudin-1, claudin-5, claudin-7 and ZO-1 were stable to acidic stress (Figure 3A). Normalized with negative controls, claudin-3 and claudin-4 displayed a significant decline followed an acidic stimulation (pH 5.0, 4.0). An approximate 26% degradation of relative quantities of claudin-3 and claudin-4 were detected by Western blot of insoluble fraction by pH 5.0 acid stimulation; more than 30% degradation by pH 4.0 acid stimulation. However, no significant changes were discovered in insoluble fraction of claudin-1, claudin-5, claudin-7 and ZO-1 (Figure 3B). The TJ proteins were visualized by cell immunofluorescence staining and confocal microscopy. As shown in Figure 4, acidification of pH 5.0 culture medium for 8 h caused reduction of claudin-3 and claudin-4, but not claudin-1, claudin-5, claudin-7 and ZO-1.

### Effects of TRPV1 and [Ca^2+^]_i_ in reduction of airway epithelial TER induced by acidification

**Figure 5:**
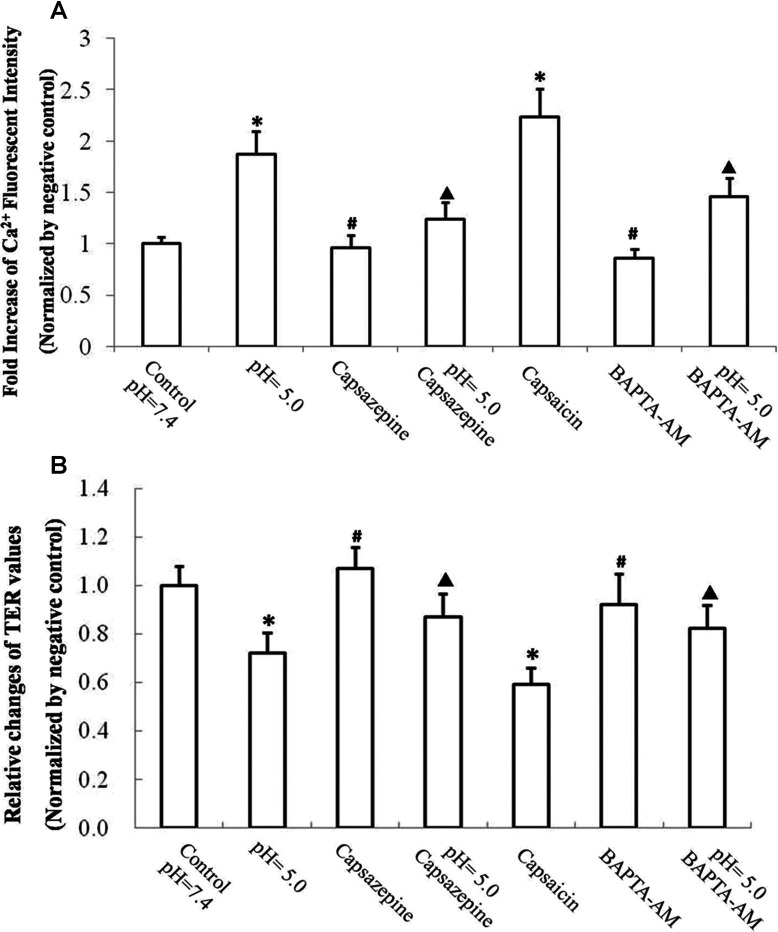
Effect of TRPV1-[Ca^2+^]_i_ pathway on impaired airway epithelial barrier function induced by acidic stress (**A**) Fluorescence intensity of Ca^2+^ in 16HBE cells after treatment of hydrochloric acid; data were normalized to the negative controls. 16HBE cells after exposure to pH 7.4 culture medium for 8 h were set as negative controls. Values are mean±S.D.; *n*=6. *P<0.05 compared with controls; ^#^*P*>0.05 compared with controls; ^▲^*P*<0.05 compared with pH 5.0 exposure group. (**B**) Relative changes of TER values in 16HBE cells after acidic stress. 16HBE cells after exposure to pH 7.4 culture medium for 8 h were set as negative controls. Values are mean±S.D.; *n*=6. *P<0.05 compared with controls; ^#^*P*>0.05 compared with controls; ^▲^*P*<0.05 compared with pH 5.0 exposure group.

To demonstrate the effect of TRPV1 non-selective iron channel on the decline of epithelial function of human airway induced by acidification, 16HBE cells were pretreated with TRPV1 antagonist capsazepine. In consideration of the pH values of EBC and the significant decline of epithelial TJs, the culture medium with the pH of 5.0 was selected for the ideal acidic stress. As previous studies described, the normal level of intracellular calcium ([Ca^2+^]_i_) maintained the stability of cell–cell TJs [24]. Selective depletion of endoplasmic reticulum calcium stores disrupts the initiation of TJs [25,26]. However, a rapid increase of [Ca^2+^]_i_ was established to be related with the loss of TJs [27,28]. To investigate the role of TRPV1 induced Ca^2+^ influx in the disruption of claudin-3 and claudin-4, 16HBE cells were pretreated with capsazepine (10 μM) and BAPTA/AM [1,2-bis-(*o*-aminophenoxy)ethane-*N*,*N*,*N*',*N*'-tetra-acetic acid tetrakis(acetoxymethyl ester)] (10 μM, a cell permeable Ca^2+^ chelator). To understand the relationship between the activation of TRPV1 and the drop of epithelial TER, 16HBE cells pretreated with TRPV1 agonist capsaicin (1 μM) were selected as a positive control. Acidification of pH 5.0 induced an approximate 2-fold increase of [Ca^2+^]_i_, which can be partly blocked by TRPV1 specific inhibitor capsazepine or BAPTA/AM (Figure 5A). Pretreatment with casazepine (10 μM) or BAPTA/AM (10 μM) exhibited a degradation of impaired TER induced by acidification of pH 5.0 culture medium (Figure 5B).

### Effects of TRPV1 and [Ca^2+^]_i_ on degradation of claudin-3 and claudin-4 induced by acidification

**Figure 6:**
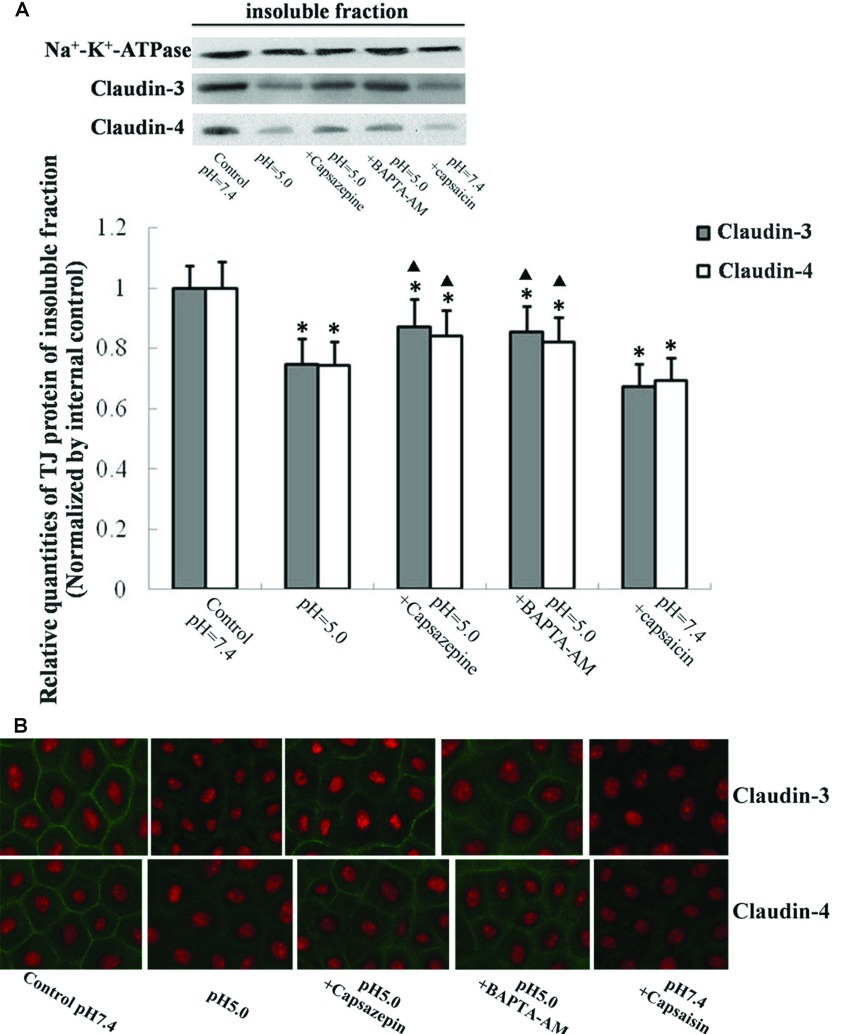
Effect of TRPV1-[Ca^2+^]_i_ on impaired airway epithelial TJ proteins (**A**) Cell extractions of insoluble fraction were loaded. 16HBE cells after exposure to pH 7.4 culture medium for 8 h were set as negative controls. Values are mean±S.D.; *n*=6. *P<0.05 compared with controls. ^▲^*P*<0.05 compared with pH=5 exposure group. (**B**) Claudin-3 and claudin-4 were visualized by immunofluorescent staining. The representative images were taken and recorded under a laser confocal microscope (original magnification, ×800).

As demonstrated by Western blot mentioned above, claudin-3 and claudin-4 were sensitive to acidic stress. To test whether the activation of TRPV1 was responsible for the degradation of claudin-3 and claudin-4 in 16HBE cells, Western blot as well as cell immunochemistry and laser confocal microscopy were used to visualize the distribution of claudin-3 and claudin-4 in 16HBE cells. Acidification of pH 5.0 culture medium caused reduction of claudin-3 and claudin-4. Pretreatment of capsaicin, the activator of TRPV1, also brought the similar effects as the acidification of pH 5.0 did. Pretreatment with capsazepine or BAPTA/AM could attenuate the reduction of claudin-3 and claudin-4 (Figure 6).

## DISCUSSION

The epithelial TJ barrier of the airway epithelium is stably maintained via the regulation of TJ molecules expressed in epithelial cells. There are several evidences about the disruption of airway TJs in airway diseases such as COPD. Morphologic changes of bronchial epithelial TJs might alter airway epithelium permeability and are associated with respiratory disorders [29]. The human airway epithelial cell line 16HBE cells we chosen in this study have a similar biological function with a similar capacity to form cell-to-cell TJs as the primary human bronchial epithelial cells [30].

The TRP family of cation channel proteins has been served as cellular sensors for detecting an array of environmental stimuli [31]. TRPV1, recognized as an acidic-sensitive member of TRP protein family, has already be detected in 16HBE cells. It has been widely accepted that TRPV1 can be uniquely activated by acidic stress or capsaicin [32]. Airway acidification is one of the symbolization of airway microenvironment in airway inflammatory diseases such as asthma and COPD. Investigations on the EBC of acute asthma drew an average pH of 5.23 [13]. Therefore an array of *in vitro* studies about acidification airway microenvironment in airway pathologies were performed on a pH at approximately 5.0 [32,33]. Previous study on the oesophageal mucosa TJs have also explored that bile acidic solutions can impair mucosal integrity [34]. According to our research data, weakly acidic stress slightly increases the TER values of 16HBE cells. However, we have not elicited a significant difference. These findings about weakly acidic stress decreasing the permeability of epithelial cells were also explored by Farre et al. [34]. The innate mechanisms about the slightly increase TER of epithelial cells under weakly acidic stress is still unclear at present. Some researchers estimated a compensatory mechanism in epithelial cells responding to weakly acidic stress, which caused a minor increase of TER [35]. In our study, we failed to draw a significant increase of TER in pH6.0 might because our study was based on monolayer culture of 16HBE, which was different from what Farre et al. did. However, higher concentration (pH=5.0, 4.0) of acid stimulation did decrease TER of 16HBE cells. We are currently researching the specific mechanism of the slight increase in TER of 16HBE cells under pH 6.0 stimulation but have been unsuccessful to date.

The activation of TRPV1 induced by acidic stress markedly increases intracellular calcium. Intracellular calcium is important for TJ integrity. Studies on the relationship between intracellular calcium and the formation of TJ indicate lowering intracellular calcium changes ZO-1/actin binding and alters the subcellular localization of occludin [36]. Excessive depletion of endoplasmic reticulum calcium stores with thapsigargin, perturbs the formation of TJs during polarized epithelial cell biogenesis [25]. Previous studies supported a critical role for intracellular calcium in TJ biogenesis in MDCK (Madin–Darby canine kidney) cells [37]. However, recent studies also suggest that rapid influx of calcium also related with the disruption of TJs. Interfering with rapid increase of intercellular calcium influx can prevent breakdown of TJs [26,27]. According to our research data, acidification by pH 5.0 culture medium caused nearly 2-fold increase of [Ca^2+^]_i_ which could be attenuated by TRPV1 selective inhibitor capsazepine. Pretreatment of TRPV1 activator capsaisin (10 μM) brought the same effect as the culture medium with the pH 5.0 did, which indicated a central role of TRPV1 in the impaired epithelial function induced by acidic stress.

The most significant finding of the our study was the novel characterization of TRPV1 non-selective cation channel dependent signalling pathway involved in epithelium barrier defect induced by acidic stress. Our data represented the first evidence that the loss of airway epithelial TJ occurred in an acidification circumstance. In spite of the slight increase of epithelial resistance induced by weakly acidic stress (pH 6.0), acidification with the pH lower than 5.0 exhibited loss of epithelial TJs as well as the epithelial resistance. The rapid increase of [Ca^2+^]_i_ via the activation of TRPV1 by acidification, resulted in the degradation of claudin-3 and claudin-4. However, claudin-1, claudin-5, claudin-7 and ZO-1 were demonstrated to be non-sensitive to acidic stress. Our work provides new horizons about the airway epithelial functions under acidic microenvironments and reveals the potential mechanisms of epithelial defect induced by acidification. TRPV1, the acid-sensitive cation channel might provide potential targets for the protection of airway epithelial function under acidic microenvironments.

## References

[1] Holgate S. T. (2007). Epithelium dysfunction in asthma. J. Allergy Clin. Immunol..

[2] Schleimer R. P., Kato A., Kern R., Kuperman D., Avila P. C. (2007). Epithelium: at the interface of innate and adaptive immune responses. J. Allergy Clin. Immunol..

[3] Gumbiner B. M. (1993). Breaking through the tight junction barrier. J. Cell Biol..

[4] Schneeberger E. E., Lynch R. D. (1992). Structure, function, and regulation of cellular tight junctions. Am. J. Physiol..

[5] Godfrey R. W. (1997). Human airway epithelial tight junctions. Microsc. Res. Tech..

[6] Wang F., Daugherty B., Keise L. L., Wei Z., Foley J. P., Savani R. C., Koval M. (2003). Heterogeneity of claudin expression by alveolar epithelial cells. Am. J. Respir. Cell Mol. Biol..

[7] Koval M. (2009). Tight junctions, but not too tight: fine control of lung permeability by claudins. Am. J. Physiol. Lung Cell Mol. Physiol..

[8] Peter Y., Comellas A., Levantini E., Ingenito E. P., Shapiro S. D. (2009). Epidermal growth factor receptor and claudin-2 participate in A549 permeability and remodeling: implications for non-small cell lung cancer tumor colonization. Mol. Carcinog..

[9] Petecchia L., Sabatini F., Usai C., Caci E., Varesio L., Rossi G. A. (2012). Cytokines induce tight junction disassembly in airway cells via an EGFR-dependent MAPK/ERK1/2–pathway. Lab. Invest..

[10] Kostikas K., Papatheodorou G., Ganas K., Psathakis K., Panagou P., Loukides S. (2002). pH in expired breath condensate of patients with inflammatory airway diseases. Am. J. Respir. Crit. Care Med..

[11] Vaughan J., Ngamtrakulpanit L., Pajewski T. N., Turner R., Nguyen T. A., Smith A., Urban P., Hom S., Gaston B., Hunt J. (2003). Exhaled breath condensate pH is a robust and reproducible assay of airway acidity. Eur. Respir. J..

[12] Koczulla R., Dragonieri S., Schot R., Bals R., Gauw S. A., Vogelmeier C., Rabe K. F., Sterk P. J., Hiemstra P. S. (2009). Comparison of exhaled breath condensate pH using two commercially available devices in healthy controls, asthma and COPD patients. Respir. Res..

[13] Hunt J. F., Fang K., Malik R., Snyder A., Malhotra N., Platts-Mills T. A., Gaston B. (2000). Endogenous airway acidification. Implications for asthma pathophysiology. Am. J. Respir. Crit. Care Med..

[14] Papaioannou A. I., Loukides S., Minas M., Kontogianni K., Bakakos P., Gourgoulianis K. I., Alchanatis M., Papiris S., Kostikas K. (2011). Exhaled breath condensate pH as a biomarker of COPD severity in ex-smokers. Respir. Res..

[15] Szili B., Bikov A., Kollai M., Horvath I. (2007). The pH of the exhaled breath condensate: new method for investigation of inflammatory airway diseases. Orv. Hetil..

[16] Oguro M., Koike M., Ueno T., Asaoka D., Mori H., Nagahara A., Uchiyama Y., Watanabe S. (2011). Dissociation and dispersion of claudin-3 from the tight junction could be one of the most sensitive indicators of reflux esophagitis in a rat model of the disease. J. Gastroenterol..

[17] Coyne C. B., Ribeiro C. M., Boucher R. C., Johnson L. G. (2003). Acute mechanism of medium chain fatty acid-induced enhancement of airway epithelial permeability. J. Pharmacol. Exp. Ther..

[18] Geppetti P., Materazzi S., Nicoletti P. (2006). The transient receptor potential vanilloid 1: role in airway inflammation and disease. Eur. J. Pharmacol..

[19] Xia R., Samad T. A., Btesh J., Jiang L. H., Kays I., Stjernborg L., Dekker N. (2011). TRPV1 signaling: mechanistic understanding and therapeutic potential. Curr. Top Med. Chem..

[20] Yu H., Li Q., Zhou X., Kolosov V. P., Perelman J. M. (2012). Transient receptor potential vanilloid 1 receptors mediate acid-induced mucin secretion via Ca2+ influx in human airway epithelial cells. J. Biochem. Mol. Toxicol..

[21] Daoui S., Cognon C., Naline E., Emonds-Alt X., Advenier C. (1998). Involvement of tachykinin NK3 receptors in citric acid-induced cough and bronchial responses in guinea pigs. Am. J. Respir. Crit. Care Med..

[22] Balkovetz D. F., Chumley P., Amlal H. (2009). Downregulation of claudin-2 expression in renal epithelial cells by metabolic acidosis. Am. J. Physiol. Renal Physiol..

[23] Gon Y., Matsumoto K., Terakado M., Sekiyama A., Maruoka S., Takeshita I., Kozu Y., Okayama Y., Ra C., Hashimoto S. (2011). Heregulin activation of ErbB2/ErbB3 signaling potentiates the integrity of airway epithelial barrier. Exp. Cell Res..

[24] Gonzalez-Mariscal L., Contreras R. G., Bolivar J. J., Ponce A., Chavez De Ramirez B., Cereijido M. (1990). Role of calcium in tight junction formation between epithelial cells. Am. J. Physiol..

[25] Stuart R. O., Sun A., Bush K. T., Nigam S. K. (1996). Dependence of epithelial intercellular junction biogenesis on thapsigargin-sensitive intracellular calcium stores. J. Biol. Chem..

[26] Brown R. C., Davis T. P. (2002). Calcium modulation of adherens and tight junction function: a potential mechanism for blood-brain barrier disruption after stroke. Stroke.

[27] Samak G., Narayanan D., Jaggar J. H., Rao R. (2011). CaV1.3 channels and intracellular calcium mediate osmotic stress-induced N-terminal c-Jun kinase activation and disruption of tight junctions in Caco-2 CELL MONOLAYERS. J. Biol. Chem..

[28] Martinez-Palomo A., Meza I., Beaty G., Cereijido M. (1980). Experimental modulation of occluding junctions in a cultured transporting epithelium. J. Cell Biol..

[29] Petecchia L., Sabatini F., Varesio L., Camoirano A., Usai C., Pezzolo A., Rossi G. A. (2009). Bronchial airway epithelial cell damage following exposure to cigarette smoke includes disassembly of tight junction components mediated by the extracellular signal-regulated kinase 1/2 pathway. Chest.

[30] Heijink I. H., Brandenburg S. M., Postma D. S., van Oosterhout A. J. (2012). Cigarette smoke impairs airway epithelial barrier function and cell–cell contact recovery. Eur. Respir. J..

[31] Samie M., Xu H., Zhu M. X. (2011). Studying TRP channels in intracellular membranes. TRP Channels.

[32] Rosenbaum T., Simon S. A., Liedtke W. B., Heller S. (2007). TRPV1 receptors and signal transduction. TRP Ion Channel Function in Sensory Transduction and Cellular Signaling Cascades.

[33] Agopyan N., Bhatti T., Yu S., Simon S. A. (2003). Vanilloid receptor activation by 2– and 10–microm particles induces responses leading to apoptosis in human airway epithelial cells. Toxicol. Appl. Pharmacol..

[34] Farre R., van Malenstein H., De Vos R., Geboes K., Depoortere I., Vanden Berghe P., Fornari F., Blondeau K., Mertens V., Tack J., Sifrim D. (2008). Short exposure of oesophageal mucosa to bile acids, both in acidic and weakly acidic conditions, can impair mucosal integrity and provoke dilated intercellular spaces. Gut.

[35] Oshima T., Koseki J., Chen X., Matsumoto T., Miwa H. (2012). Acid modulates the squamous epithelial barrier function by modulating the localization of claudins in the superficial layers. Lab Invest..

[36] Ye J., Tsukamoto T., Sun A., Nigam S. K. (1999). A role for intracellular calcium in tight junction reassembly after ATP depletion-repletion. Am. J. Physiol..

[37] Stuart R. O., Sun A., Panichas M., Hebert S. C., Brenner B. M., Nigam S. K. (1994). Critical role for intracellular calcium in tight junction biogenesis. J. Cell Physiol..

